# What Does Galvanic Vestibular Stimulation Actually Activate: Response

**DOI:** 10.3389/fneur.2012.00148

**Published:** 2012-10-22

**Authors:** Bernard Cohen, Sergei B. Yakushin, Gay R. Holstein

**Affiliations:** ^1^Departments of Neurology, Mount Sinai School of MedicineNew York, NY, USA; ^2^Departments of Neuroscience, Mount Sinai School of MedicineNew York, NY, USA; ^3^Department of Anatomy/Functional Morphology, Mount Sinai School of MedicineNew York, NY, USA

Curthoys and MacDougall (2012) question the conclusion that galvanic vestibular stimulation (GVS) predominantly activates the otolith system. They have demonstrated that strong (5 mA) GVS applied continuously to alert humans in darkness causes weak nystagmus, with average velocities of about 5°/s (MacDougall et al., 2002, 2003, 2005). This nystagmus undoubtedly originated from activation of the semicircular canals. They claim that this refutes our general conclusion that the predominant activation of the vestibular system during GVS is through the otolith system, a conclusion based on a wide range of studies in which GVS induced a sense of roll, ocular counter-torsion, postural sway, and body tilt but not nystagmus, vertigo, or a sense of rotation. The results were uniform across these diverse studies, although some were conducted in darkness or with the eyes closed. They also question conclusions based on an anatomic demonstration by Holstein et al. (2012) that c-fos activation was essentially located in otolith-driven, vestibulo-sympathetic neurons in the vestibular nuclei (Holstein et al., 2012). Curthoys has made many important contributions to elucidating semicircular canal and otolith anatomy and physiology, but we believe that he and MacDougall are not correct in this issue.

Important evidence for our conclusion derives from a compendium of studies by Macefield and colleagues showing that sinusoidal GVS is a potent stimulus for induction of muscle sympathetic nerve activity (MSNA); a reflection of otolith activation. In these studies, currents of 2 mA induced a sense of rolling but never rotation or vertigo (Bent et al., 2006; Grewal and James, 2009; James and Macefield, 2010; James et al., 2010; Hammam et al., 2011, 2012; Grewal et al., 2012). The sense of roll is consistent with a host of other studies using GVS (Fitzpatrick et al., 1994; Inglis et al., 1995; Day et al., 1997; Zink et al., 1997; Day and Cole, 2002; Scinicariello et al., 2002/2003; Wardman et al., 2003a,b; see Fitzpatrick and Day, 2004 for review). Modeled on this research, we used 2–3 mA currents in lightly anesthetized rats and found strong activation of the sympathetic nervous system and vasovagal responses, but not tonic deviations of the eyes (Cohen et al., 2011). We have also stimulated the vestibular nerve with trains of pulses to activate MSNA in humans (Voustianiouk et al., 2006), sometimes utilizing currents of 5 mA. MSNA was facilitated, but ocular deviations were never induced.

The semicircular canals can exert a powerful influence on eye movements through the vestibulo-ocular reflex. Head turns induce eye movements with velocities of up to 400°/s (Atkin and Bender, 1968), and with response characteristics up to 20 Hz (Grossman et al., 1988; Tabak and Collewijn, 1994; Armand and Minor, 2001). Furthermore, a change in body temperature of only 7°C during caloric stimulation with either air or water readily induces nystagmus with slow phase velocities of 30–40°/s in monkeys (Arai et al., 2002) and 10–20°/s in humans (M. Dai, personal communication). Thus, at best, the semicircular canal activation induced using strong GVS by Curthoys and MacDougall, which produced average slow phase eye velocities of 5°/s, was weak and functionally inconsequential.

The question of function is particularly relevant in this regard. The semicircular canals stabilize gaze in space during head movement through what is probably the fastest reflex in the body. That is, electrical stimulation of canal nerves can activate eye muscles in 1.5 ms, which would potentially allow for a frequency response of 600 Hz (Cohen and Suzuki, 1963). In actuality, eye muscles were driven at 400 Hz and prominent nystagmus with large beats and high velocity were induced by semicircular canal nerve stimulation (Cohen et al., 1965). The canals also activate neck muscles at short latencies to help stabilize the head in space (Denise et al., 1987; Xiang et al., 2008). On the other hand, the otolith organs, while having a small direct ocular response to sharp increases in linear acceleration of the head (Paige and Tomko, 1991), do not produce nystagmus and there is only weak ocular torsion of about 4° from vestibular nerve stimulation that orients the eyes to the spatial horizontal in frontal-eyed species, including humans. Thus, the otolith system appears not to be primarily directed toward controlling eye movements in humans, although one can get small rapid eye movements from rapid head movements (Curthoys, 2010). Rather, the otolith system appears to be more involved in orientation in space, in activation of the vestibulo-sympathetic reflex, and in stabilization of posture, none of which are functions of the semicircular canals.

Therefore we conclude that our previous reply to Dr. Colebatch and our original letter in which we stated that while there may be weak semicircular canal activation, the response to GVS is predominantly otolithic in function are correct, and we do not find the Curthoys and MacDougall argument sufficiently compelling to refute our conclusion.
